# Evidence against the “anomalous-is-bad” stereotype in Hadza hunter gatherers

**DOI:** 10.1038/s41598-022-12440-w

**Published:** 2022-05-24

**Authors:** Clifford I. Workman, Kristopher M. Smith, Coren L. Apicella, Anjan Chatterjee

**Affiliations:** 1 Department of Neurology, University of Pennsylvania, Philadelphia, PA 19104 USA; 2 Penn Brain Science Center, University of Pennsylvania, Philadelphia, PA 19104 USA; 3 Penn Center for Neuroaesthetics, University of Pennsylvania, Philadelphia, PA 19104 USA; 4 Department of Anthropology, Washington State University, Pullman, WA 99163 USA; 5 Department of Psychology, University of Pennsylvania, Philadelphia, PA 19104 USA

**Keywords:** Psychology, Human behaviour, Social evolution

## Abstract

People have an “anomalous-is-bad” stereotype whereby they make negative inferences about the moral character of people with craniofacial anomalies like scars. This stereotype is hypothesized to be a byproduct of adaptations for avoiding pathogens. However, evidence for the anomalous-is-bad stereotype comes from studies of European and North American populations; the byproduct hypothesis would predict universality of the stereotype. We presented 123 Hadza across ten camps pairs of morphed Hadza faces—each with one face altered to include a scar—and asked who they expected to be more moral and a better forager. Hadza with minimal exposure to other cultures chose at chance for both questions. Hadza with greater exposure to other cultures, however, expected the scarred face to be less moral and a better forager. These results suggest the anomalous-is-bad stereotype may be culturally shared or learned erroneously through associations with population-level differences, providing evidence against a universal pathogen avoidance byproduct hypothesis.

## Introduction

If Hollywood is to be believed, then people are usually only as *good* as they are good looking. In contrast to heroes, movie villains are more frequently depicted with visible facial anomalies, like scars, that negatively impact perceptions of attractiveness^1^. In the *Star Wars* universe, for instance, Anakin Skywalker’s transformation into Darth Vader is marked by the simultaneous corruption of his moral character and of his physical form. The notion that facial appearance weighs on character is more than just a familiar storytelling device—participants from primarily English-speaking countries have a “beauty-is-good” stereotype and are more likely to ascribe positive personal qualities to beautiful people^2–5^. On the other hand, US students and online workers have an “anomalous-is-bad” stereotype and are more likely to ascribe negative qualities to people with deviations from prototypically attractive features (e.g., craniofacial anomalies), especially negative attributions of moral character^5–8^.

People may use craniofacial anomalies and other deviations from facial typicality to infer moral character because these anomalies are perceived as cues to infectious disease. Facial characteristics associated with beauty on the other hand, like symmetry and averageness, may serve as cues of healthiness that prospective partners can use to assess mate quality^9^. US students with more attractive faces have better in vitro immunological responses to bacterial and viral infections^10^. Consistent with the foregoing evolutionary account, preferences for symmetry and averageness appear to be universal. The Hadza hunter-gatherers of Tanzania show preferences for symmetrical Hadza and European faces and for more average Hadza faces (though—perhaps owing to a lack of familiarity—not for more average European faces)^11,12^. Of note, although some prior work has suggested a more specific link between facial symmetry and pathogen avoidance, a recent registered report casts doubt on such a link^13^. Humans have an evolved behavioral immune system to identify and avoid potential vectors of pathogens, using cues that were historically associated with increased pathogen risk, such as coughs, sneezes, lesions, and bodily fluids^14^. Failing to detect threats of pathogens can have costly consequences, whereas vigilance against such threats is likely to be less costly, even when triggered erroneously by irrelevant cues^15–17^. In conditions of uncertainty, the behavioral immune system errs on the side of caution by biasing behavior towards vigilance in order to limit the harmful consequences of potential errors^16,17^.

Cues of pathogen threat may include facial anomalies, even when such anomalies are acquired independently of any underlying illnesses^18^. Indeed, facial anomalies and unattractive faces are treated as expressions of infectious disease that elicit disgust and avoidance in perceivers^19–21^. Moreover, expressions of negative bias in attitudes and behavior towards people with facial anomalies are predicted by disgust-sensitive brain responses to anomalous faces^6–8^, further evidence that disgust underpins reactions to anomalous faces and that people erroneously infer pathogen threat from anomalies^18^. Abundant cross-cultural evidence suggests that feelings of disgust motivate the avoidance of both pathogen threats and moral threats^22–27^. People express and report disgust toward pathogen-irrelevant actions, such as unfairness, dishonesty, violence, and child abuse^28–32^, and stronger experiences of disgust toward violations are associated with increased moralistic punishment^23,25,33^. Children as young as 6, for instance, reportedly responded with disgust more often for moral violations (e.g., meanness directed at another person) than for negatively valenced yet morally neutral actions (e.g., watching a sad movie with another person)^25^. Finally, people also infer negative moral character from experiences of disgust, which motivates the avoidance of immoral others^34,35^.

If the anomalous-is-bad stereotype is a byproduct of adaptations designed to detect and avoid potential pathogen threats, then it should be expressed broadly across cultures, which requires testing in diverse samples^36^. However, most of the evidence for the stereotype comes from Western populations, which are outliers on many psychological dimensions compared to the global population^37,38^. Existing evidence on perceptions of trustworthiness from facial appearance suggests variation of the stereotype between and within cultures. For example, Dutch^39^, US, and Japanese^40^ adults vary in the extent to which they believe traits can be inferred from facial features (i.e., lay physiognomy beliefs), and people with stronger beliefs in physiognomy are more likely to form character impressions using facial appearance. Further, the extent to which people infer trustworthiness and dominance from facial appearance varies across cultures^41^. While these studies did not examine the anomalous-is-bad stereotype cross-culturally, they provide evidence that specific impressions formed from facial appearance are not universal.

**Figure 1:**
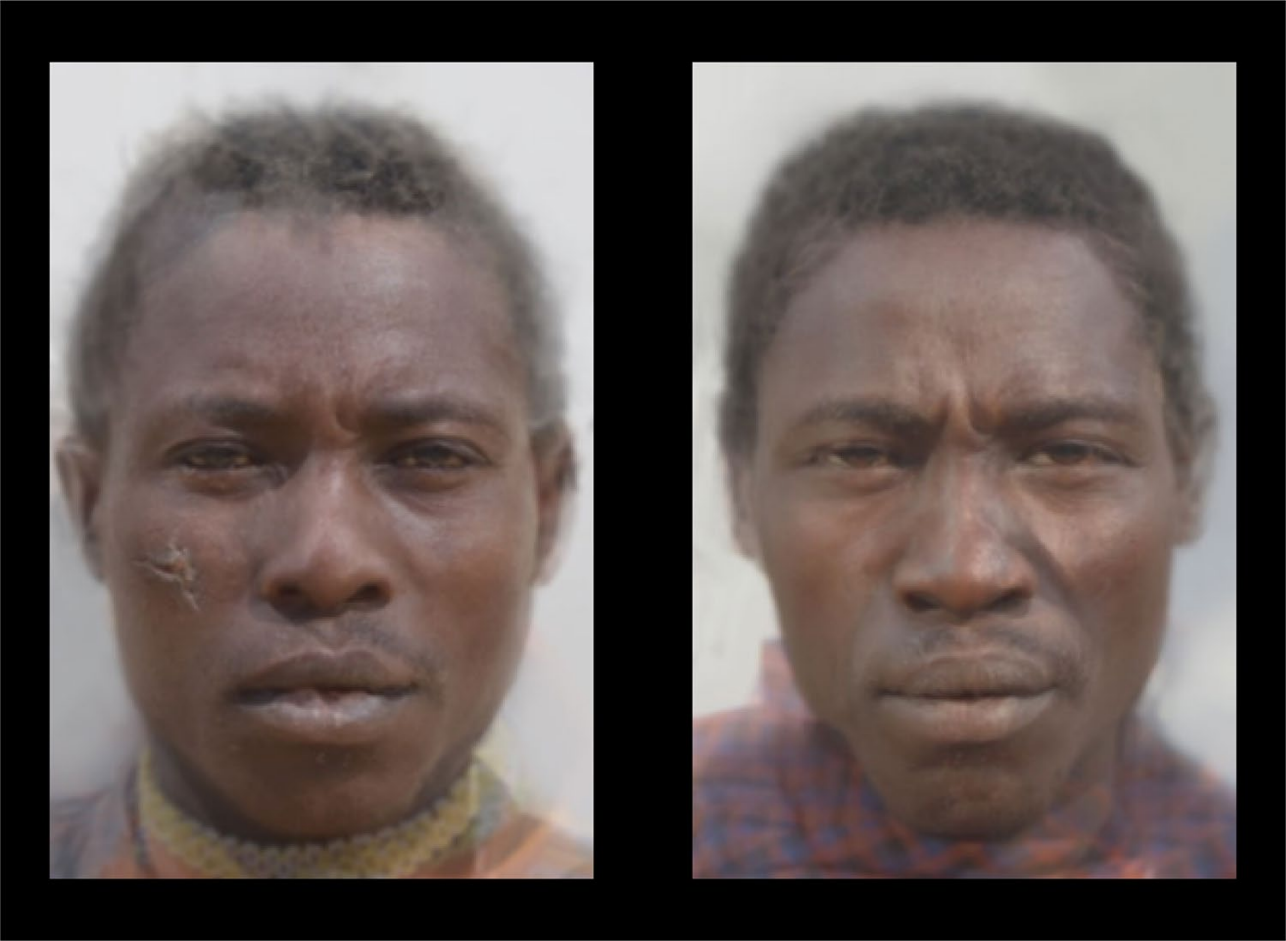
A sample set of composite male faces used in the experiment (face set M1, condition A). The complete set of stimuli are available at https://osf.io/eqftk/.

Here, we examine the anomalous-is-bad stereotype among the Hadza. The Hadza are not isolated, and have had contact since at least the nineteenth century with neighboring populations, including Datoga, Isanzu, Sukuma, and Maasai^42^; however, they have had minimal contact with Western media, such as television, magazines, and cinema. In addition, the Hadza practice intentional scarification, making small vertical or lateral incisions on the cheeks with a small knife. The function of these incisions is thought to be manifold: they serve the aesthetic purpose of marking children as “Hadza,” and they allow for the application of medicines when treating injuries and ailments^42–44^. Intentional scarification for purposes of beautification and rites of practice are common in East Africa^45^. Intentional scarification appears to place limits on claims about the universality of the anomalous-is-bad stereotype. Scars acquired *intentionally* may function as signals of robust health and pathogen resistance that enhance attractiveness^46^ with no such benefit conferred to unintentional scars. As such, the Hadza may not draw the same inferences from scarred faces as Western participants. We asked participants to choose between pairs of faces—one with and one without a superimposed scar (see Fig. 1)—and asked them to indicate which face appeared more moral and which appeared to be a better forager. If the Hadza are no less likely to choose the scarred face, then this would be evidence that the anomalous-is-bad stereotype is not universal. We ask about morality and foraging ability because some evidence suggests that the stereotype is specific to morality^5^, and foraging ability is an important dimension on which Hadza evaluate campmates^47,48^. Asking about foraging ability thereby allows us to examine the specificity of the stereotype. Finally, we also explore variation within the Hadza as a function of exposure to non-Hadza cultures. Exposure to non-Hadza cultures is associated with economic biases^49^, emotional closeness to other ethnic groups^50^, greater sharing in an economic game^51^, and preferring to live with more generous campmates^47^. Exposure may similarly moderate whether and how they infer character qualities about other Hadza.

## Results

Table 1 presents the proportion of responses choosing the anomalous face by face set, condition, and trait. The only difference between conditions was which face in the pair had the facial anomaly (see Method for details). Before presenting estimates from the model, two patterns are noticeable in the raw data. First, collapsed across all face sets, participants were more likely to choose the anomalous face for good forager, and this pattern was observed within all but one face set. Second, collapsed across all face sets, participants were less likely to choose the anomalous face for good heart, and this pattern was observed in five of the eight face sets and conditions.

**Table 1:** Proportion of responses choosing anomalous face by face set, condition, and trait.

Face set	Condition	Good forager	Good heart
F1	A	0.39	0.34
	B	0.59	0.54
F2	A	0.52	0.28
	B	0.50	0.59
M1	A	0.63	0.45
	B	0.54	0.43
M2	A	0.60	0.39
	B	0.54	0.52
Total		0.54	0.43

Numeric values are the proportion of responses that chose the anomalous face for that trait. Face sets with women are notated with “F” and face set with men are notated with “M.” The only difference between conditions was which face had the photoshopped scar.

Table 2 presents the estimates from the marginal posterior distribution for each population parameter. When considering who is a better forager, the probability of preferring the anomalous face was positive; further, the effect of exposure on the probability of choosing the anomalous face as a better forager was positive. When considering who has a better heart, the probability of preferring the anomalous face was negative; further, the effect of exposure on the probability of choosing the anomalous face as having a better heart was negative.

**Table 2:** Parameter estimates from the marginal posterior distributions of the model.

Parameter	Median	90% HDI	*pd*	% in ROPE
Good forager	0.25	− 0.03, 0.54	0.932	33.1
Good heart	− 0.27	− 0.59, 0.05	0.921	29.6
Good forager × Exposure	0.13	− 0.03, 0.30	0.910	68.3
Good heart × Exposure	− 0.13	− 0.29, 0.04	0.913	69.7

The point estimate is the median value of the marginal posterior distribution. The 90% highest density interval (HDI) is the smallest interval that contains 90% of the posterior distribution, *pd* is the proportion of the posterior in the same direction as the median, and % in ROPE is the proportion of the posterior distribution falling between the region of practical equivalence (− 0.18).

We next examined the joint posterior prediction distribution and whether, across the population, participants preferred the non-anomalous face for each trait, collapsing across participant exposure, face, and condition. The probability of preferring the anomalous face as a better forager was greater than chance, *Med.* = 0.57, 90% HDI: 0.48–0.68, *pd* = 0.899. The probability of preferring the anomalous face as having a better heart was less than chance, *Med.* = 0.42, 90% HDI: 0.31–0.52, *pd* = 0.900.

**Figure 2:**
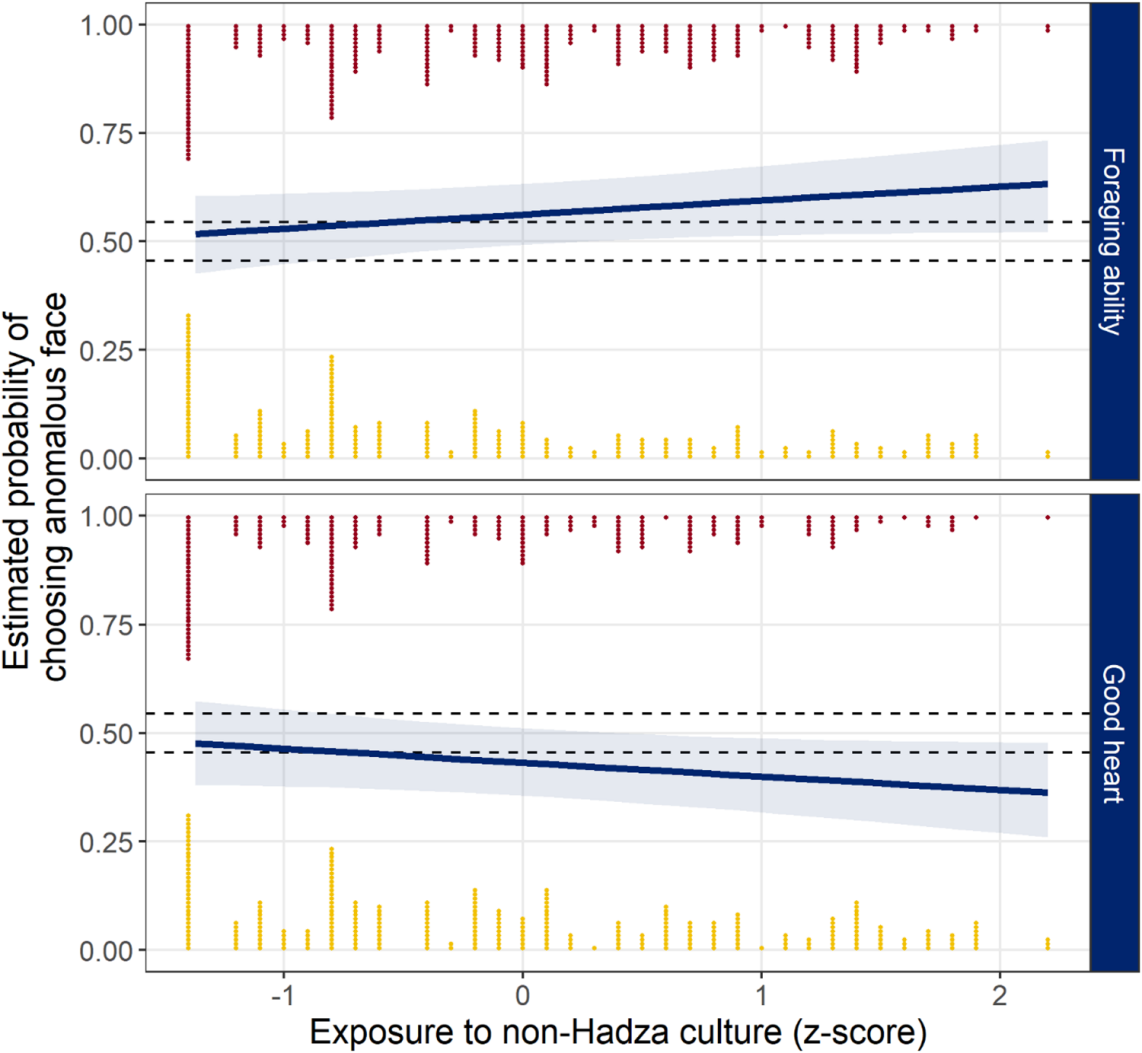
Joint posterior predictions by participants’ exposure to non-Hadza culture as a *z*-score and trait being evaluated. Lines are the median estimate from the posterior and shaded regions are the 90% highest density intervals (HDI). Points are individual responses (for plotting, exposure values were rounded to the nearest tenth), with red points at the top indicating when the participant chose the anomalous face, and the yellow points at the bottom indicating when the participant chose the non-anomalous face. Dashed lines indicate the region of practical equivalence (ROPE).

Finally, we examined the joint posterior prediction distribution by participants’ exposure to other cultures. Examining participants with the lowest exposure, the probability of preferring the anomalous face as a better forager was about chance, *Med.* = 0.52, 90% HDI: 0.43–0.61, *pd* = 0.630, 57.3% in ROPE, and similarly the probability of preferring the anomalous face as having a better heart was about chance, *Med.* = 0.48, 90% HDI: 0.38–0.57, *pd* = 0.660, 53.0% in ROPE. However, for participants with the highest exposure, the probability of preferring the anomalous face as a better forager was greater than chance, *Med.* = 0.63, 90% HDI: 0.53–0.74, *pd* = 0.972, 8.9% in ROPE, whereas the probability of preferring the anomalous face as having a better heart was less than chance, *Med.* = 0.36, 90% HDI: 0.26–0.47, *pd* = 0.972, 8.8% in ROPE. Figure 2 presents the estimated probabilities as a function of exposure to non-Hadza culture for each trait.

### Analyses by same-sex and opposite-sex judgments

**Figure 3:**
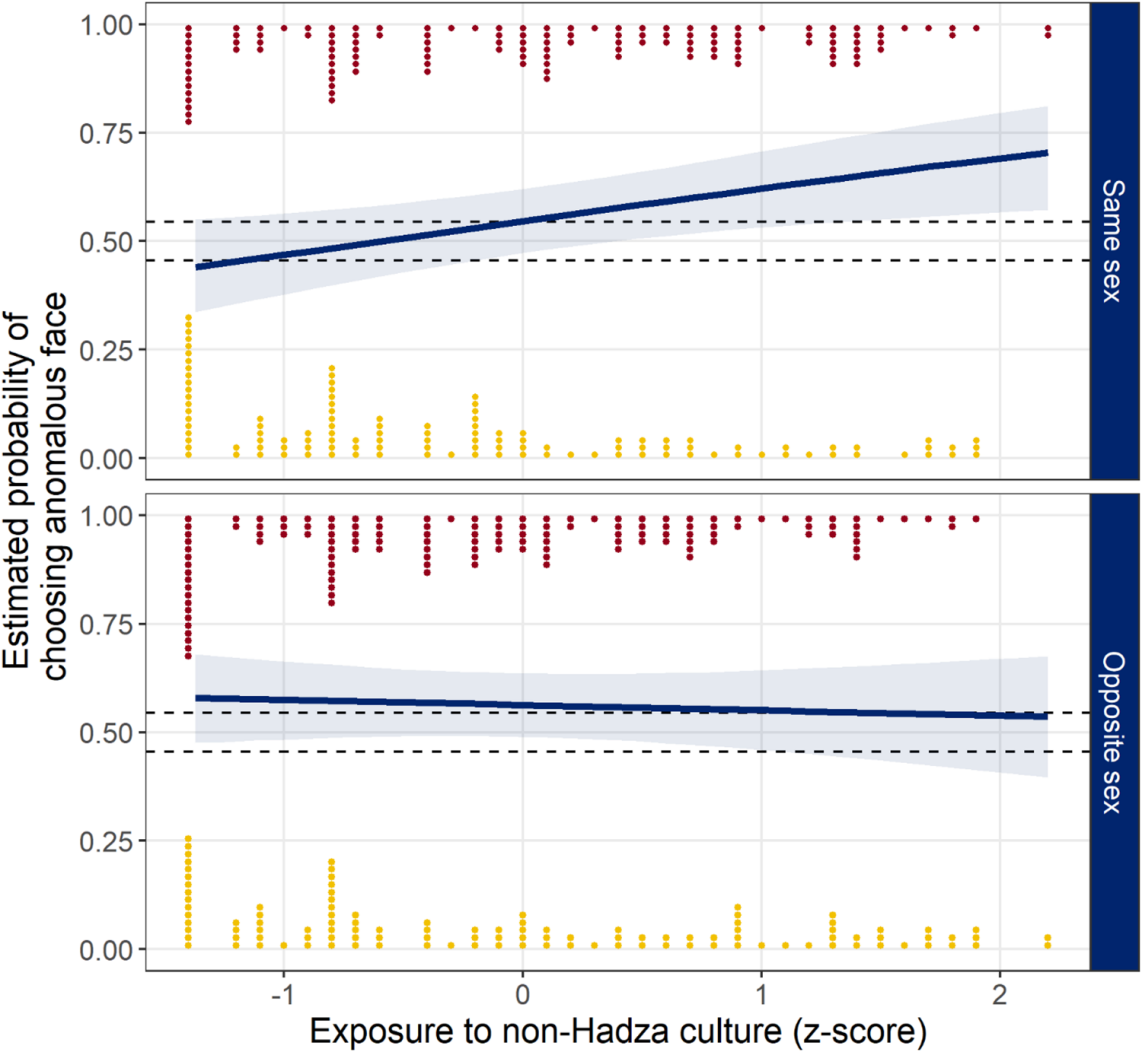
Joint posterior predictions of choosing the anomalous face for good forager judgments by participants’ exposure to non-Hadza culture as a *z*-score and whether the face was same- or opposite-sex. Lines are the median estimate from the posterior and shaded regions are the 90% highest density intervals (HDI). Points are individual responses (for plotting, exposure values were rounded to the nearest tenth), with red points at the top indicating when the participant chose the anomalous face, and the yellow points at the bottom indicating when the participant chose the non-anomalous face. Dashed lines indicate the region of practical equivalence (ROPE).

We analyzed the data in a second model including an interaction with whether the participant was judging a face that was the same-sex or opposite-sex. See Table 3 for parameter estimates from the model. When judging which face has a better heart, participants with greater exposure were more likely to choose the non-anomalous face, regardless of whether the judgments were of same- or opposite-sex faces. However, when judging which face is a better forager, preferences differed between same- and opposite-sex judgments. When judging faces of the same-sex, participants with greater exposure were more likely to choose the anomalous face as a better forager, but when judging faces of the opposite-sex, participants were slightly more likely to choose the anomalous face as a better forager, regardless of exposure (see Fig. 3 for predicted probabilities of choosing anomalous faces for good forager judgments).

**Table 3:** Parameter estimates from the marginal posterior distributions of the model analyzing same- and opposite-sex judgments.

Parameter	Median	90% HDI	*pd*	% in ROPE
**Same-sex judgments**				
Good forager	0.19	− 0.12, 0.48	0.852	46.8
Good heart	− 0.21	− 0.54, 0.14	0.846	41.2
Good forager × Exposure	0.31	0.07, 0.53	0.989	17.8
Good heart × Exposure	− 0.13	− 0.35, 0.09	0.830	63.9
**Opposite-sex judgments**				
Good forager	0.25	− 0.05, 0.55	0.921	32.4
Good heart	− 0.30	− 0.65, 0.02	0.926	26.4
Good forager × Exposure	− 0.05	− 0.27, 0.17	0.640	79.3
Good heart × Exposure	− 0.12	− 0.37, 0.08	0.823	64.4

The point estimate is the median value of the marginal posterior distribution. The 90% highest density interval (HDI) is the smallest interval that contains 90% of the posterior distribution, *pd* is the proportion of the posterior in the same direction as the median, and % in ROPE is the proportion of the posterior distribution falling between the region of practical equivalence (− 0.18).

## Discussion

People in English-speaking populations have a beauty-is-good stereotype, inferring that people perceived as more attractive are more moral and that people perceived as physically unattractive are less moral^3,5^. People with deviations from facial typicality, such as craniofacial anomalies like scars, are especially likely to be viewed as morally worse, whether they are perceived as attractive or not. In other words, people have an anomalous-is-bad stereotype^6^. We found evidence that this stereotype is not culturally universal. Hadza hunter-gatherers with minimal exposure to other cultures were not more likely to think other Hadza with facial scarring were less moral or worse foragers. However, Hadza who regularly interact with outside cultural groups were more likely to think Hadza with facial scarring were less moral and were better foragers. These results suggest the anomalous-is-bad stereotype is culturally shaped, providing evidence against the hypothesis that the stereotype is a universal byproduct of the behavioral immune system.

Cultural exposure may moderate character inferences among the Hadza through the acquisition of beliefs from neighboring populations about what constitutes facial attractiveness. Previous research finds that culture influences attractiveness judgments. For example, South African Zulu migrants to the UK rated women with a lower body-mass index and waist-to-hip ratio as more attractive compared to Zulus residing in South Africa^52^. South Korean students with greater exposure to US media are more likely to rate thin models as attractive^53^. Hadza find more average Hadza faces more attractive, but not more average white European faces, suggesting exposure to different faces is necessary to judge average faces as more attractive^12^. Some Hadza may have learned from neighboring populations that facial scars are unattractive and consequently infer negative character traits from facial scarring. Alternatively, Hadza could believe facial scarring is unattractive but, through exposure to non-Hadza culture, acquire beliefs linking specific traits with attractiveness. US, Japanese, and Dutch participants who do not believe traits can be inferred from facial features are less likely to make such inferences^39,40^, providing direct evidence that explicit beliefs can influence the social evaluation of faces. Some Hadza may similarly learn associations between moral character and attractiveness from neighboring populations. One possibility is that Hadza with exposure to non-Hadza culture learn that facial scars bear negatively on perceptions of attractiveness, which then has downstream negative consequences for judgments of moral character, instead of learning that scars bear directly on moral character. This is an interesting prospect, but is at odds with the finding in this study that scars also have positive consequences for judgments of foraging ability, as better foragers are considered more attractive^54^.

Exposure to non-Hadza cultures could also moderate the moral character concept among Hadza. In Western populations, intent to do harm or to commit a moral violation is central to moral character judgments^55,56^. Intention to do wrong suggests a desire to commit the act, which is used to infer the potential to commit moral violations again in the future. That is, intention suggests the moral character concept is stable among Western particpants^57^. However, there is variation across populations in the extent to which intentions influence moral judgments, and many small-scale populations, such as the Hadza, Himba, and Yesawa, do not condemn intentional actions more than unintentional actions^58,59^. Further, Hadza disagree on perceptions of who is more moral^48^ and do not infer moral character from others’ decisions in response to sacrificial dilemmas^60^, suggesting they may not think morality is a stable characteristic of a person. If so, then moral character would not be associated with other relatively stable traits, such as facial appearance. However, as Hadza become more exposed to other cultures, their moral character concept may change, rendering them more willing to infer character from physical characteristics.

Alternatively, culture may play a less direct role in forging associations between scars and perceptions of moral character. It is possible that, instead of internalizing the “anomalous-is-bad” stereotype, Hadza with greater exposure to non-Hadza culture may be perceived as more prestigious among the Hadza, having greater education and access to markets. Hadza may also infer that people with unintentional scars are spending more time in the bush and less time in villages. Hadza have higher levels of fluctuating asymmetry compared to European and North American populations^61^, possibly because of high rates of accidents Hadza experience while foraging^44,62^. If true, then Hadza may also infer that people with scars have less exposure and lower status, and Hadza with greater exposure—and thus greater prestige—negatively judge Hadza with anomalous faces as lower status. Across cultures, valuations of social and moral standing are correlated^63^, and Hadza with greater exposure may also judge lower-status Hadza as less moral. We do not have the data to explore this hypothesis in the current study, though addressing this and the alternatives presented above more directly is a goal of future research.

Surprisingly, participants with greater exposure to non-Hadza culture thought Hadza with scars were better foragers. Prior studies report that people make negative attributions from physical unattractiveness for a broad range of traits, including competence^3,8^, whereas others report effects specific to moral traits^5^. This finding could be an experimental artifact because foraging judgments were always asked after good heart judgments. Participants could have avoided selecting the same faces for both traits, such that picking the unscarred face for the good heart judgment would likely be followed by picking the scarred face for foraging. However, in previous studies, Hadza do not have difficulty discriminating between pairs of faces presented multiple times^11,12^. The significance of exposure to non-Hadza culture for judgments of foraging ability is further complicated by the sexes of the raters and of the faces being judged. Similar to what was observed without accounting for sex, participants with more exposure were more likely to think same-sex anomalous faces belonged to better foragers but, regardless of exposure, all participants chose the anomalous opposite-sex faces as better foragers. Consistent with evidence that facial scars may serve as indicators of beauty in some small-scale African societies, the presence of scars on opposite-sex Hadza faces may have positively influenced their perceived attractiveness with especially potent consequences for foraging judgments^45,54^. If so, however, such a positive effect would have also been expected for moral character judgments instead of the negative effect we observed. Another explanation is that accidents and injuries happen somewhat frequently during foraging bouts^44^. Hadza with facial scars may look more “bush-like” to other Hadza, who then interpret scars as indicators of foraging ability. Further research is needed to unpack the significance of exposure, sex, and attractiveness to judgments of foraging ability.

Do we assume people with facial anomalies are villains because they are portrayed as such in movies, or are villains portrayed with facial anomalies because of a pre-existing association between morality and beauty? Our data suggest that culture shapes the inferences people draw from physical appearance. Specifically, we find that the anomalous-is-bad stereotype is not expressed universally and is largely absent among Hadza without exposure to other cultures. However, there is variation within the Hadza as a function of their exposure. This study provides evidence against the hypothesis that the anomalous-is-bad stereotype is a byproduct of adaptations to avoid pathogens. The contrast between these findings and earlier work that detected the anomalous-is-bad stereotype in Western populations highlights the importance of including more diverse populations in person perception research.

## Method

### Population

The Hadza are a population of nomadic hunter-gatherers living in northwestern Tanzania. About 1,000 people today speak Hadzane and identify as Hadza, but only approximately 300 still obtain a majority of their calories from foraged goods^44,62^. Hadza live in temporary residence bands called camps, with about 20–30 adults and children in each camp. Camps usually consist of about two to three unrelated nuclear families^62^. Camps move location every six to eight weeks as local resources are consumed, and any individual can move freely to another camp at any time. Each year, individual Hadza interact with about one-fifth of the foraging population^64^, and from year to year, Hadza live with approximately one-in-five of their previous campmates^65^. The Hadza are highly interdependent with their campmates, and all food brought back to camp— especially meat—is shared with all other members of the camp^44^. Like other hunter-gatherer populations, the Hadza have a sexual division of labor, in which men hunt for large game and collect honey, while women collect plant goods such as tubers and berries^44^.

### Sample

KMS and a team of research assistants visited 10 camps in September and October 2019. Camps were sampled using a snowball sampling procedure—after finishing interviews at one camp, someone in the camp would direct the research team to the next nearest camp. There was no predetermined sample size. The team sampled camps until no more camps could be located, and interviewed 126 adults, with three exclusions for cognitive impairments that prevented participants from understanding the task. The final sample was 123 adults (60 women, 89 married) with a mean age of *M* = 37.6 (*SD* = 14.2) years. Each participant made eight decisions in the experimental task for a total of 984 observations in our analyses.

### Procedure

#### Forced-choice task

Participants were presented with four pairs of same-sex faces that were composite photographs representing novel Hadza. In each pair, one face had visible scars while the other had no scarring. After looking at each pair, participants chose the person they believed better exemplified a given trait. Participants were first asked which person in the photograph had a better heart, which is a judgment of global moral character^48^. Participants indicated their choice by pointing at the photograph. After answering for all four pairs, participants were again presented each pair and asked which man was a better hunter, and which woman was a better gatherer. Participants always saw the pairs in the same order, with the two male pairs presented first. The questions were also asked in the same order each time. Before their interviews, participants were randomly assigned to a condition by coin flip. The only difference between conditions was which face in each pair had visible scarring. This part of the interview was conducted in Hadzane by a Hadza research assistant and overseen by a Tanzanian research assistant.

#### Survey of exposure to other cultures

We estimated the extent to which each participant had been exposed to non-Hadza cultures using their knowledge of and experience with the surrounding cultural groups. Specifically, participants reported the number of years they attended school, whether they had worked a job, how high they could count in Swahili, if they had visited Arusha or lived outside of Hadzaland, if they knew the capital of Tanzania, and whether they knew the Tanzanian president, Barack Obama, Nelson Mandela, and Mahatma Gandhi. A two-factor structure best fit the data with responses to all but the questions about international figures loading better on to the first factor. A standardized composite score was computed using the weighted sums from the first factor as a measure of exposure to other cultures^47^. This part of the interview was conducted in Swahili by a Tanzanian research assistant and overseen KMS.

### Stimuli

The experimental stimuli were constructed using 2D headshot photographs acquired from actual Hadza as part of an earlier study^48^. Consenting adult Hadza were photographed in 2016 with a Fujifilm Instax Mini 90 Classic Instant Film Camera. To standardize the photographs, participants were—whenever possible—positioned in front of a portable screen and instructed to adopt an emotionally neutral facial expression.

A total of 32 face photographs (16 male, 16 female) were pre-processed using established procedures:^6,66^ First, the photographs were normalized to inter-pupillary distance with algorithms from the OpenCV computer vision library (https://opencv.org/) and facial landmarks from the dlib machine learning toolkit (http://dlib.net/). Second, they were resized and cropped in IrfanView (https://irfanview.com/; width: 380px; height: 570px) and then edited automatically (remove.bg, https://www.remove.bg/) and then manually (GIMP 2, https://www.gimp.org/) to remove backgrounds.

After pre-processing, each of the 32 face photographs was submitted to the InterFace software package, used to place landmarks at 82 fiducial points on each face^67^. The resulting coordinates were converted to JPsychoMorph format^68^. Then, JPsychoMorph’s averaging feature was used to generate 16 composite images (8 male, 8 female) derived from subsets of 4 male or female faces from the complete set of 32 photographs. This required a total of 64 photographs (16 composites × 4 faces), so all 32 photographs were used twice (always in different composites). Next, visual inspection was used to identify and eliminate 8 composites that looked unrealistic owing to averaging errors. This resulted in a set of 8 useable composites (4 male, 4 female).

Finally, copies of the 8 composites were manually edited in Adobe Photoshop. Specifically, images of real bodily scars identified through web searches were carefully superimposed onto copies of all 8 composites. The final set of stimuli was 16 composites of four male and female faces, with an anomalous and non-anomalous version of each.

### Data analysis and computation

We analyzed participants’ decisions in Bayesian multilevel regression models^69^. We modeled the choice as a Bernoulli function with probability *p* of choosing the anomalous face; logit(*p*) was a function of the trait the participant was being asked about, the participant’s exposure to other cultures and its interaction with the trait in question. We specified the model not to estimate an intercept and instead estimated each effect as a difference from responding at chance, with separate estimates for each trait. We included varying slopes of each trait for participant and camp. In addition, for face set, we included varying slopes for each condition, each trait, and their interactions. This approach effectively estimates which face for each set was more likely to be picked for each trait. All varying effects were modeled as multivariate normal distributions. Priors were weakly regularizing priors centered around zero. Prior predictive checks verified that all but the most extreme of values (e.g., choosing the anomalous or non-anomalous face with > 95% probability) were plausible under our priors.

In the analyses, we present marginal posterior distributions of the population parameters and posterior distributions of predicted probabilities. For the distribution of predicted probabilities, we compute expected probability of choosing the anomalous face from trait, exposure, face set, and condition. We then collapse estimates across variables not of interest for the analysis to get a marginal posterior of the predicted values. We use the median of the distribution as a measure of the central tendency, 90% highest density intervals (HDI) as a measure of uncertainty, probability of direction (*pd*) as a measure of existence of an effect, and percent of the posterior in the region of practical equivalence (ROPE) as a measure of significance^70^. HDI is the smallest continuous interval of values that 90% of the posterior falls between, *pd* is the proportion of the posterior above (or below depending on the sign of the median) zero (for parameter values) or 0.50 (for predicted probabilities), and % in ROPE in the proportion of the posterior distribution that falls within a predefined region around zero (or 0.50). Existence is the probability there is an effect in the direction indicated by the median, whereas significance (as indicated by the inverse of the percent in ROPE) is the probability that the effect is practically different from zero^71^. We defined the ROPE as the interval of − 0.18–0.18 following guidelines for a “small” effect for standardized coefficients in logistic regression^70,72^.

We conducted the analyses in R (4.1.1), using the brms package (2.15.0)^73^ with cmdstanr (0.4.0)^74^ to fit the model, the tidyverse (1.3.1)^75^ package for data wrangling and visualization, and the tidybayes (3.0.0)^76^ and modelr (0.1.8)^77^ packages for summarizing posterior distributions and predictions. We fit each model with two chains using within-chain parallelization, with 10,000 iterations each, 5,000 of which were for warmup, for a total of 10,000 samples post-warmup. Visual inspection showed that that the chains were well-mixed, and for every parameter the effective number of samples in the bulk and tail of the distributions was > 2,000, indicating effective sampling of the posterior.

### Transparency and ethics

The study design and data analysis plan were not preregistered. Materials, de-identified data, R scripts, and statistical outputs are publicly available at https://osf.io/eqftk/. Study protocols were approved by the IRB at the University of Pennsylvania and permission to conduct research in Tanzania was approved by the Tanzanian Commission for Science and Technology. All methods were performed in accordance with the guidelines and ethical standards set out by relevant national and institutional committees on human experimentation and with the Helsinki Declaration of 1975, as revised in 2008. Permission to work in a camp was granted by each camp as a group and verbal informed consent to participate in research from each participant was obtained individually.
